# A Rare Case of Dirofilariasis in the Genian Region

**DOI:** 10.3390/diagnostics14060628

**Published:** 2024-03-15

**Authors:** Andrei Nicolau, Florin Petrică Sava, Florentina Severin, Mihai Liviu Ciofu, Dan Ferariu, Daniela Dodu, Victor Vlad Costan

**Affiliations:** 1 Department of Oral and Maxillo-Facial Surgery, “Grigore T. Popa” University of Medicine and Pharmacy, 700115 Iasi, Romania; nicolau.andrei@umfiasi.ro (A.N.); mihai.ciofu@umfiasi.ro (M.L.C.); victor.costan@umfiasi.ro (V.V.C.); 2 Department of ENT, “Grigore T. Popa” University of Medicine and Pharmacy, 700115 Iasi, Romania; 3 Department of Pathology, Regional Institute of Oncology, 700483 Iasi, Romania; d_ferariu@yahoo.com; 4 Department of Radiology, Clinical Hospital of Rehabilitation, 700661 Iasi, Romania; dodudaniela@yahoo.com

**Keywords:** dirofilariasis, *Dirofilaria repens*, parasitic cyst, genian region

## Abstract

Dirofilariasis is an infectious disease caused by species of the *Dirofilaria* genus. It is manifested by the appearance of a subcutaneous swelling, especially in the eye region. We present the case of a 29-year-old patient who presented with facial asymmetry in the right genian region. Following clinical and paraclinical evaluations, the diagnosis of a parasitic cyst was established in the context of dirofilariasis with *Dirofilaria repens* (*D. repens*). Treatment consisted of surgical excision of the formation associated with prophylactic antibiotic medication. Macroscopic analysis of the excision piece revealed a structure that contained a cystic cavity and a filamentous form with a length of approximately 10 mm and a diameter of 1 mm. This is the first case of dirofilariasis located in the genian region reported in Romania. The overview of this pathology is important to raise awareness among physicians about its presence and clinical variations. Understanding such cases helps healthcare professionals enhance diagnostic skills, refine treatment strategies, and provide valuable insights into the prevalence and clinical presentation, fostering early detection and timely intervention. Detailed case reports contribute to the understanding of the disease’s epidemiology, including risk factors and transmission patterns, which is essential for effective public health strategies.

## 1. Introduction

The World Health Organization (WHO) has reported that more than 120 million people are affected by filariasis, a mosquito-borne disease [1]. *Dirofilaria immitis* and *Dirofilaria repens* are the main causative agents of dirofilariasis disease and subcutaneous dirofilariasis [2]. Transmission of *D. repens*, occurs mainly through mosquito bites, particularly by the *Aedes caspius* mosquito [3].

*D. repens* is a vector-borne filaroid helminth of canids, with dogs representing the major reservoirs of infestation [4]. The full life-cycle of *D. repens* consists of five larval stages with a latent period of 6 to 9 months [5]. The development of the parasite is conditioned by numerous factors: the availability of competent mosquito species, suitable hosts, adult male and female *D. repens* helminths, and the presence of the bacterial endosymbiont, Wolbachia [6], which is required for the successful molting and embryogenesis of filariae. *D. repens* is a type of subcutaneous nematode, which, even though characteristic of dogs and cats, can also be transmitted to humans, causing an infectious condition called dirofilariasis. Humans acquire *D. repens* infestation in the same manner as dogs after the bite of a mosquito species from the *Culicidae* family [7].

In most cases, infective larvae are detected by the body’s immune system, leading to destruction of the parasite before the infestation is diagnosed [8]. In some cases, the larva can survive and molt into a pre-adult and adult worm.

This infection is generally located in the upper areas of the body, but can also affect the male and female genitals, lungs or other internal organs [9]. *D. repens* infestation is characterized by local inflammation, mainly in subcutaneous and ocular tissues with symptoms that are usually mild and resolve immediately after surgical extraction of the worm [10].

The development of *D. repens* into a mature nematode in humans is uncommon. Antigen sets from both *D. repens* and their endosymbiont Wolbachia stimulate specific immunologic reactions that block the complete development of the nematode [5], and for this reason, humans are considered to be dead-end hosts [11]. In rare cases, *D. repens* can avoid the host’s defense mechanisms and reach maturity [12]. In the literature, there are currently 11 case reports of human *D. repens* microfilaremia, and only a few have been confirmed with molecular analysis [13].

Dirofilariasis caused by *D. repens* is more common in the ocular area, but the parasite has the ability to migrate to other regions of the body. It is estimated that its migration speed is about 30 cm in just two days, and previous research has reported significant migration between different parts of the body [14]. 

It is crucial to raise awareness among healthcare professionals about infections caused by *Dirofilaria* species [15]. Surgeons might initially mistake dirofilariasis as a malignancy if they are unfamiliar with this infection, which could cause significant distress to patients. To accurately diagnose dirofilariasis, it is important to possess knowledge regarding the typical location and migration patterns of the nematode, take into account the patient’s travel history, and recognize the clinical symptoms associated with the infection [16]. Corroborating the clinical examination with ultrasonography, an imaging method that identifies changes in the cystic content and the position of the intracystic filamentous structure, heightens suspicion of a parasitic cyst. As the sole clinical site, a clearly defined lesion, characterized by the presence of a capsule and the cyst’s dimensions, presents the challenge of meticulous surgical excision without exposing the cystic content and potential parasitic structure.

Differentiation of *Dirofilaria* species from other nematodes is primarily based on morphological analysis, which includes the use of light microscopy, scanning electron microscopy and/or DNA-based analysis [17]. Diagnosis of this condition is usually established by microscopic and macroscopic examination of the nematode or by histopathological analysis, which reveals the presence of a thick, multilayered cuticle [18]. In addition, high-resolution ultrasound imaging proves useful in detecting parasite movement within subcutaneous nodules. Treatment of subcutaneous dirofilariasis usually requires excision of the nematode or surgical removal of the nodule [19].

In the literature, few cases of parasitic cysts caused by *D. repens* located in the zygomatic region have been reported [1]. Our report presents an unusual clinical case of a patient diagnosed with a cystic outgrowth of parasitic origin in the right genian region in the context of dirofilariasis disease caused by *D. repens*, treated in the Oral and Maxillo-facial Surgery Clinic of the “St. Spiridon’’ Emergency Clinical Hospital of Iasi, Romania. The significance of this particular case is primarily represented by the location of the cyst in the genian region, which leads to several differential diagnoses for the clinician and contributes significantly to the existing literature by drawing attention to this location and avoiding diagnostic errors. This adds valuable insights to the diverse manifestations of dirofilariasis, potentially influencing diagnostic protocols and therapeutic approaches specific to cases involving the head and neck region.

## 2. Case Report

**Figure 1:**
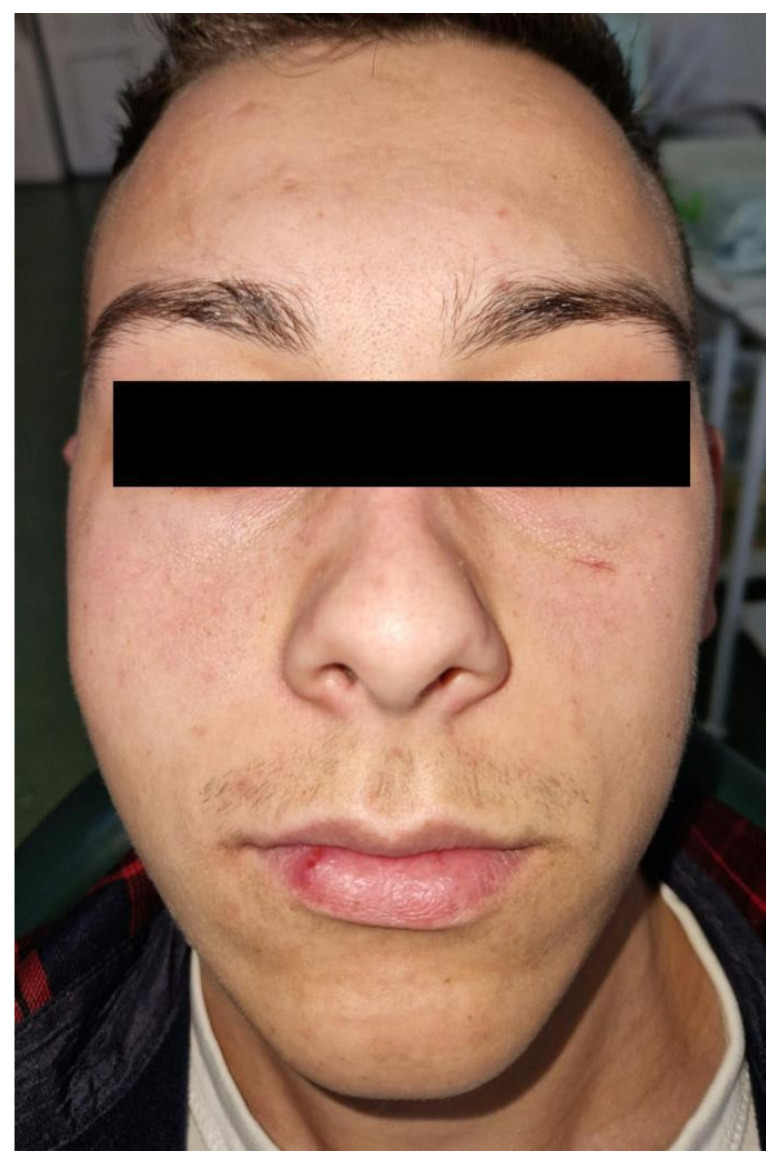
Facial asymmetry in the right genian region.

A 29-year-old male patient, a farm worker with no medical history, was hospitalized in the Clinic of Oral and Maxillo-facial Surgery of “St. Spiridon” Emergency Clinical Hospital of Iasi, Romania, for the diagnosis and treatment of a facial asymmetry localized in the right genian region (Figure 1). The patient declared that the swelling had a slow growth without producing sensitive alterations of the infraorbital nerve or functional disorders. The patient could not specify any past facial trauma.

**Figure 2:**
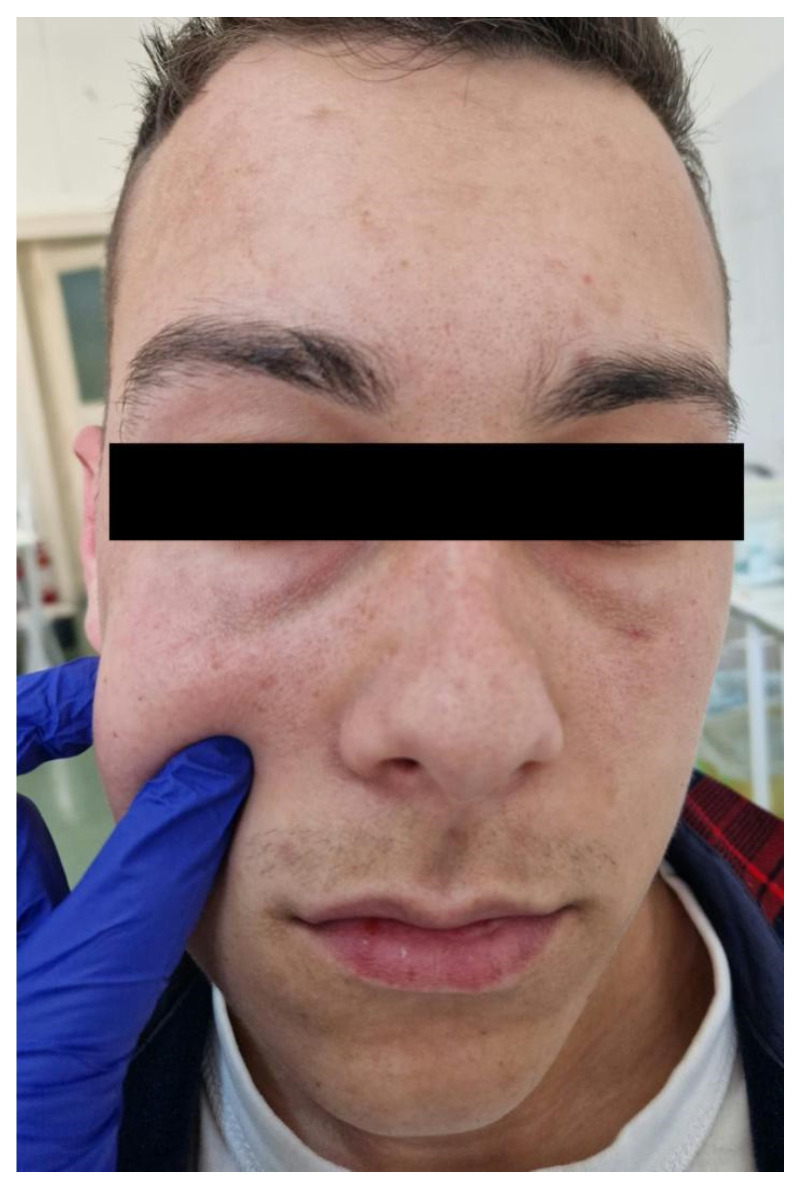
Clinical evaluation of the swelling.

To evaluate the swelling located in the right genian region, a clinical examination associated with ultrasound investigation was performed. The outgrowth was covered by normal and unmodified skin. Through clinical examination, an oval, mobilizable and painless formation with a diameter of 18/15/10 mm with a firm–elastic consistency was identified (Figure 2). 

No cervical or general lymphadenopathy was detected. The complete blood count (CBC) had no modifications besides a mild thrombocytopenia with no clinical significance (Table 1).

**Figure 3:**
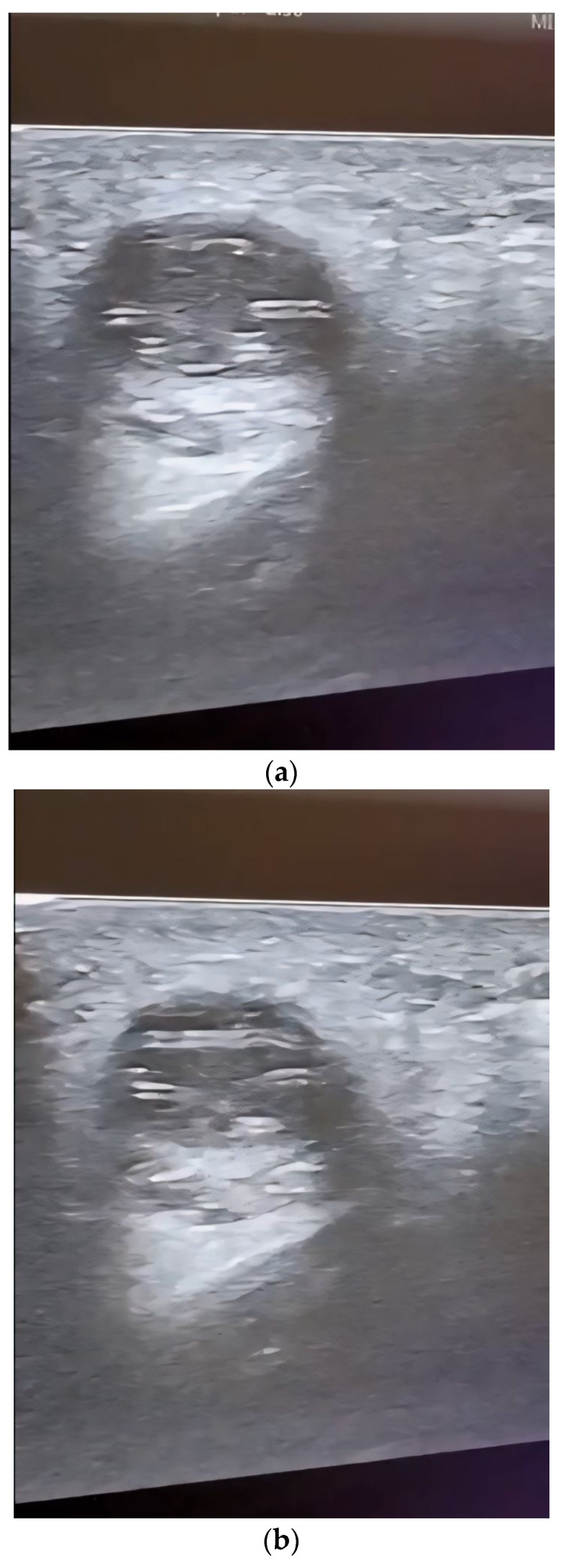
(**a**,**b**) Ultrasound image of the genian region showing a cystic mass including a motile tubular structure in different positions: (**a**) position 1; (**b**) position 2.

**Figure 4:**
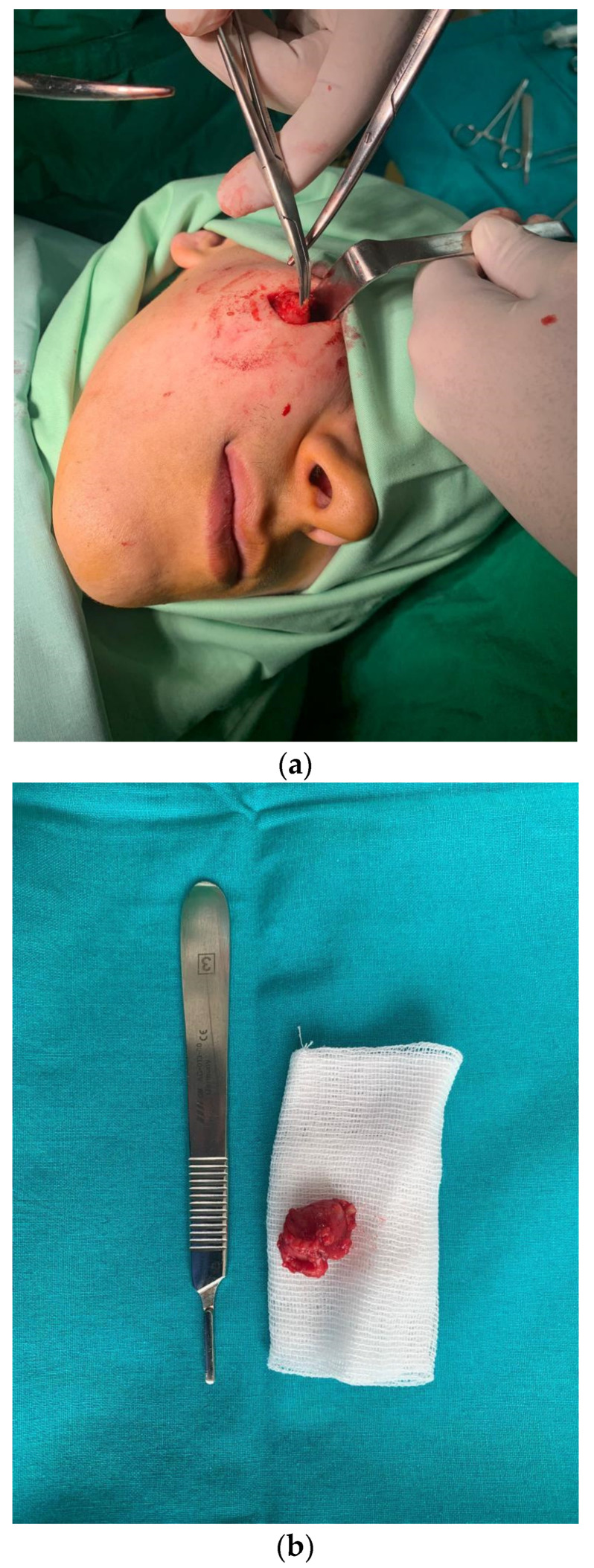
(**a**) Surgical excision of the parasitic cyst; (**b**) the excised unaltered formation.

Through ultrasound scanning of the genian region, we detected a well-defined round hypoechogenic lesion that included a moving tubular structure with a length of 10 mm and a width of 1 mm (Figure 3 and Figure 4).

No further tests for circulating microfilariae were performed. Following the clinical and ultrasound examination, the surgical team decided to remove the outgrowth through classical surgical treatment; thus, an incision was made, followed by the excision of the entire cystic formation without it being damaged (Figure 4a,b).

**Figure 5:**
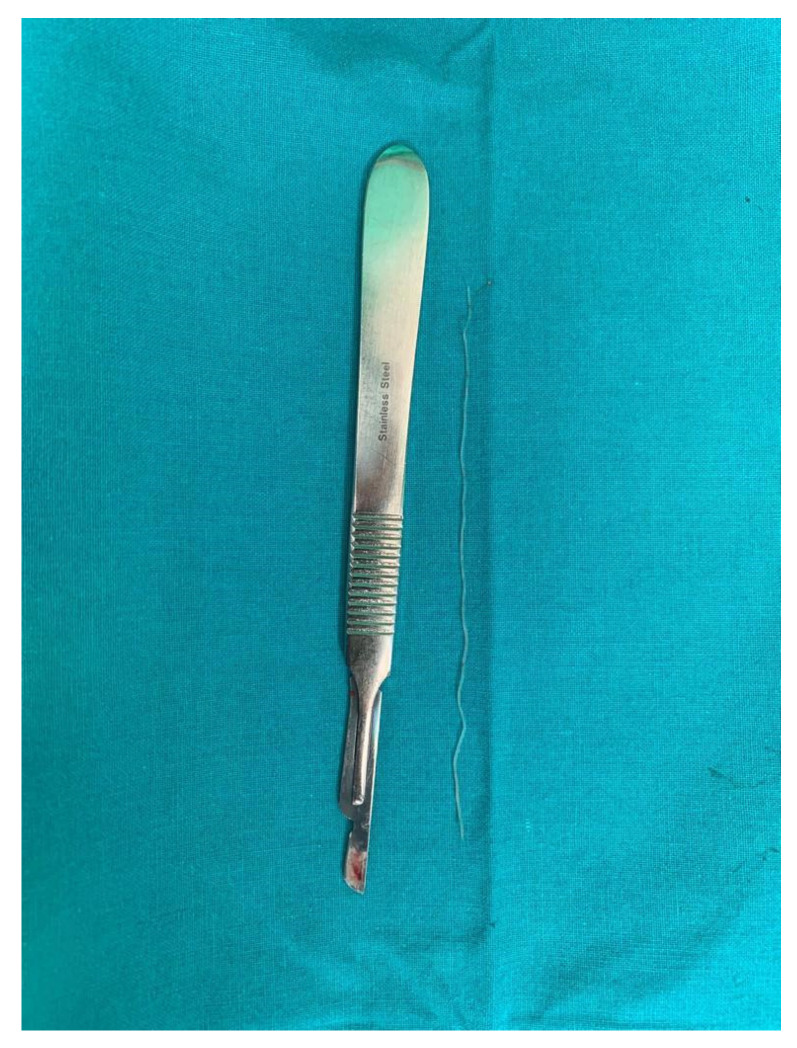
Images of the *D. repens* parasite extracted from the cystic cavity.

Histological examination of the excision piece revealed, at the macroscopic level, a tissue fragment of 18/15/10 mm, with a subtotal cross-section, in which a cystic cavity of approximately 7 mm in the large axis was found, containing a filamentous structure of approximately 10 mm in length and 1 mm in thickness (Figure 5).

**Figure 6:**
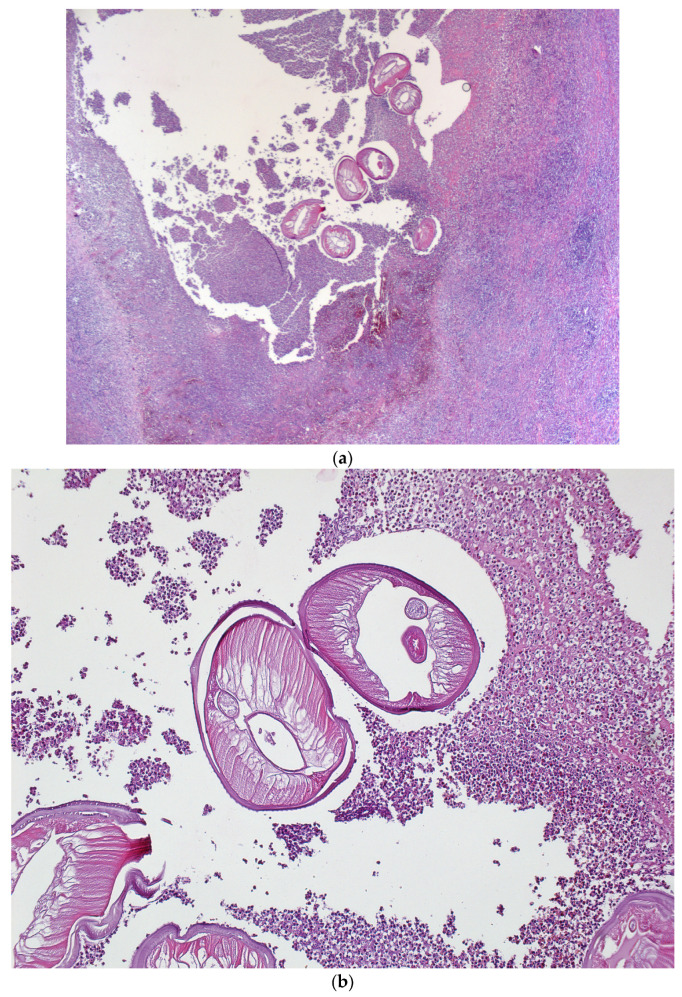
Hematoxylin and eosin staining; (**a**) ×25, abscess cavity with multiple transverse sections through the parasite; (**b**) ×100, *D. repens*—cuticle discretely scalloped, striated.

Microscopically, histological sections with hematoxylin and eosin staining evidentiated an abscess with a wall consisting of maturing granulation tissue with peripheral fibrosis, and polymorphous inflammation associated with lymphocytes, plasmocytes, eosinophils, polymorphonuclears and histiocytes, some multinucleated, could be observed. The macroscopically observed filamentous structure was represented by a nematode mark in the periphery by a cuticle with a discrete scalloped relief (Figure 6).

From a morpho-pathological point of view, the morphological aspects correspond to a parasitic cyst in the context of dirofilariasis disease, with the external structure of the parasite suggesting *D. repens*.

Following the surgical excision, only prophylactic antibiotherapy was given, considering that the surgical excision was performed on the soft tissues of the face, the cystic outgrowth was completely removed, and the capsule was not perforated or interrupted during the surgical excision. No specific antiparasitic medication was necessary considering it was the only localization with no other signs and symptoms after the surgical excision.

The postsurgical wound evolved without any complications and underwent full healing. The sutures were removed 7 days after the surgery.

## 3. Discussion

Human nematode infections, although not very common, show an increasing trend; WHO statistics show that they affect approximately 90 million people worldwide [1].

*D. repens* is a nematode that is localized subcutaneously, found especially in dogs and cats in Europe, Africa and Asia, and can be transmitted to humans through mosquito bites [20]. 

All filarial nematodes of medical and veterinary importance rely on *Wolbachia* symbiosis, with the exception of Loa loa [21]. The species of the genus *Wolbachia* are Gram-negative members of the *Alphaproteobacteria* that belong to the order *Rickettsiales* [22].

*Wolbachia* infects filarial nematodes and many insects, including some mosquito species. *Wolbachia* is required for the development and survival of filarial nematodes [23], whereas its symbiotic relationship with mosquitoes is largely parasitic [24]. *Culicidae* mosquito species are known to be infected with *Wolbachia* and are considered to be vectors for *Dirofilaria* [25,26,27,28,29].

The genome sequencing of both Wolbachia and *D. repens* revealed that the nematodes have become dependent on their endosymbionts for a diverse range of biological processes [30], such as the synthesis of metabolites including haem, riboflavin, flavin adenine dinucleotide and nucleotides, which are provided by Wolbachia to the nematode, which cannot synthesize these molecules de novo [31].

Adults and microfilariae can cause diseases that are sometimes lethal in animals and extremely debilitating in humans [32]. The nematodes live in the rich fluids of their hosts (lymph, blood), and most nutrients are acquired through the hypodermis rather than the digestive tract [32]. *Wolbachia* are found in great abundance in this tissue, which represents a hot spot for worm metabolism. The hypodermis delivers nutrients to sustain gametogenesis and the huge demand of embryonic development takes place entirely in the female uterus, occupying almost the entire length of the female body [22].

*Wolbachia* plays a significant role in the desensitization of host innate immunity, assuring the nematode’s long-term survival [33]. No inflammatory reaction or connective tissue capsule surrounds the living parasite, which can be seen moving actively under the connective serous layers [34,35]. In most cases, the infection has no clinical signs [34,35]. Cutaneous disorders such as pruritus, dermal swelling, subcutaneous nodules containing the parasite, and ocular conjunctivitis can be present [36]. In our case, the subcutaneous parasitic cyst was the only clinical manifestation. Allergic reactions, likely due to microfilaria sensitization and Wolbachia-mediated inflammatory reactions in severe infections, have been reported [37]. In these cases, circulating microfilariae are often absent.

The infection has a predominant localization in the upper areas of the body with a percentage of 75.8%, but also in the male and female genital organs with a percentage of 6.5% or in the female breast in 5.4% of cases [1,20]. Among the infections appearing in the upper part of the body, 30.5% have been reported as being localized in the eye region, but due to the movable character of the parasite, its location can change [1,38].

Dirofilariasis parasites are transmitted by insects such as *Aedes caspius* mosquitoes, which have the ability to travel long distances and bite during the entire day [1,39]. One study showed that 1.5% of this mosquito species contains *D. repens* DNA [40]. A previous paper reported the interesting cases of some patients who presented with the existence of a nematode under the skin, and the initial diagnosis was of illusory parasitosis, with the presence of a movable nematode proven later [39]. The infection with the parasite *D. repens* can be manifested by the appearance of subcutaneous swelling with slow evolution, located predominantly in the eye area [20]. Due to its localization and its slowly evolving and painless character, it can be confused with a benign tumor such as an adenoma, mucocele or lipoma [41]. The clinical signs and evolution of the parasitic cyst in our case limited the differential diagnosis to benign tumors arising from the soft tissues of the face. Malignancy was overruled by the long evolution, the very well-delimited formation that did not infiltrate the surrounding tissues, and the lack of lymphadenopathy.

The ultrasound examination’s role in the diagnosis was essential for our case, revealing the filamentous structure moving inside the encapsulated mass from the right genian region, orientating the diagnosis immediately towards a parasitic cyst. 

The diagnosis of dirofilariasis is performed by a serological, fluorescent antibody test and the ELISA technique, and recently the polymerase chain reaction, but it is most commonly given by the pathologist after an excisional biopsy.

Laboratory results of complete leukocyte count, relative lymphocyte and eosinophil count, Knott’s test and molecular detection of *D. repens* and *Wolbachia*, prior to and after surgical extraction of nematodes, should be performed in all patients with dirofilariasis [4].

A modified Knott’s test performed on 6 mL of EDTA blood (6 × 1 mL) [4] is used to reveal the presence of circulating microfilaria and should be performed in all patients with dirofilariasis. In order to provide a more accurate insight into the number of patients with circulating microfilariae infected with *D. repens*, the modified Knott’s test should be used regularly and with larger blood volumes than 1 mL [4]. In our case, no other laboratory tests were performed besides the complete blood count, which showed no modifications in leukocyte and eosinophil count. A rise in the number of white blood cells and of the eosinophils can be present in some cases. In a literature review, 11 cases of dirofilariasis had microfilaremia, representing 50% of the cases included in the study [4].

Identifying the nematode histologically can raise some challenges for the pathologist, mainly as dirofilariasis is not among the common pathologies encountered. In some cases, an advising opinion from a pathologist with expertise in parasitic diseases is necessary.

This paper reports the case of a 29-year-old patient with no general medical conditions presenting a painless swelling in the right genian region secondary to infection with the parasite *D. repens*. Following the analysis of the literature, it can be affirmed that this is the first case of a parasitic cyst caused by *D. repens* infection localized in the genian region reported in Romania [42,43,44,45].

Treatment against dirofilariasis is exclusively surgical, consisting of the removal of the cystic outgrowth along with the parasite. Previous articles report a failure rate of surgical treatment of 5% [41,46]. Preventing the transmission of dirofilariasis involves controlling mosquito vectors and administering prophylactic treatments. Mosquito control measures, such as eliminating breeding sites and using insecticides, can reduce transmission. Prophylactic treatments involve administering preventive medications, like monthly heartworm preventives for pets, which target the immature stages of *Dirofilaria*. Combining both strategies is crucial for effective prevention. Regular veterinary check-ups and compliance with preventive medications are key components for pets.

In the case of migratory parasitic cysts, the administration of anthelmintic drugs is recommended to reduce the motility of the nematode and to create a fixed structure that can be removed [16].

Although in other case reports, patients infected with *D. repens* stated that prior to contact with the parasite they had traveled to areas such as Croatia, France, Italy, Russia or Sri Lanka, in our case report, the patient had not traveled in recent years and had not reported any trauma in the antecedents [5,15,17,47,48].

## 4. Conclusions

The presented case report demonstrates that dirofilariasis can be located in other areas outside the eye region. Dirofilariasis caused by *D. repens* should also be suspected in geographic regions that are not considered endemic as long as a patient presents the specific symptoms. Surgical excision of the parasitic cyst and *D. repens* is sufficient to ensure healing. This case report underscores that dirofilariasis may manifest beyond the ocular region, emphasizing the need to consider *D. repens* even in non-endemic areas when patients exhibit relevant symptoms. While surgical excision proves effective for healing, future research could explore diagnostic improvements, such as non-invasive methods or molecular techniques, and investigate optimal postoperative care protocols to enhance patient outcomes. Additionally, assessing the prevalence of *D. repens* in diverse geographic regions would contribute to a more comprehensive understanding and guide clinical practices.

## Figures and Tables

**Table 1:** Complete blood count.

WBCs	5.05 × 10^3^/µL
RBCs	4.5 × 100^3^/µL
Hb	14.4 g/dL
Platelets	145 × 10^3^/µL
Neutrophils	3.11 × 10^3^/µL
Lymphocytes	1.26 × 10^3^/µL
Monocytes	0.61 × 10^3^/µL
Eosinophils	0.06 × 10^3^/µL
Basophils	0.02 × 10^3^/µL

## Data Availability

Data are contained within the article.
